# Identifying and costing common gaps in Central and West Africa pharmaceutical regulation

**DOI:** 10.3389/fmed.2024.1362253

**Published:** 2024-04-05

**Authors:** Claudia P. Alfonso, Guy B. N’Jambong, Alaa Magdy, Laura Di Trapani, Rutendo Kuwana, Abraham G. Kahsay, Diadié Maïga, Sybil N. A. Ossei-Agyeman-Yeboah, Aimé B. Djitafo Fah, Margareth Ndomondo-Sigonda

**Affiliations:** ^1^Regulatory Systems Strengthening, World Health Organization, Geneva, Switzerland; ^2^Essential Drug and Medicines, World Health Organization, Dakar, Senegal; ^3^ICN Business School, CEREFIGE, Université de Lorraine, Nancy, France; ^4^Incident and Substandard and Falsified, World Health Organization, Geneva, Switzerland; ^5^Regulatory Convergence and Networks, World Health Organization, Geneva, Switzerland; ^6^West Africa Health Organization, Bobo-Dioulasso, Burkina Faso; ^7^Sub-Regional Program for the Harmonization of National Pharmaceutical Policies in Central Africa, Coordination Organization for the Fight Against Endemic Diseases in Central Africa, Yaoundé, Cameroon; ^8^African Medicines Regulatory Harmonization Initiative, African Union NEPAD Agency, Pretoria, South Africa

**Keywords:** regulatory systems strengthening, national regulatory authorities, common regulatory needs, institutional development plan, substandard and falsified medical products, good regulatory practice, quality management systems, public health emergencies

## Abstract

**Background:**

Regulatory systems strengthening is crucial for catalyzing access to safe and effective medical products and health technologies (MPHT) for all. Identifying and addressing common regulatory gaps through regional approaches could be instrumental for the newly incepted African Medicine Agency.

**Aims:**

This original study sheds light on common gaps among 10 national regulatory authorities (NRAs) and ways to address them regionally.

**Objectives:**

The study used NRA self-assessment outcomes to identify common gaps in four critical regulatory pillars and estimate the cost of addressing them from regional perspectives that aimed at raising the maturity level of regulatory institutions.

**Methods:**

A cross-sectional study, using the WHO Global Benchmarking Tool (GBT), was conducted between 2020 and 2021 with five NRAs from ECCAS and ECOWAS member states that use French and Spanish as *lingua franca*.

**Results:**

The 10 NRAs operated in a non-formal-to-reactive approach (ML1-2), which hinders their ability to ensure the quality of MPHT and respond appropriately to public health emergencies. Common gaps were identified in four critical regulatory pillars—good regulatory practices, preparedness for public health emergencies, quality management systems, and substandard and falsified medical products—with overall cost to address gaps estimated at US$3.3 million.

**Contribution:**

We elaborated a reproducible method to strengthen regulatory systems at a regional level to improve equitable access to assured-quality MPHT. Our bottom-up approach could be utilized by RECs to address common gaps through common efforts.

## 1 Introduction

Strengthening regulatory systems that support equitable access to medical products and health technologies (MPHT) is vital for continuous improvement in health outcomes and achievement of Universal Health Coverage (UHC) (1–6). Understanding inequities in access to MPHT is crucial for improved health policy that addresses the needs of low- to middle-income countries (LMICs) (7–9) and stronger regulatory systems for medicines (10). Through the regulatory systems strengthening (RSS) program, WHO works with Member States (MS) and financial and technical partners to improve access to assured-quality MPHT (11–14). Since 2017, among the NRAs in 13 MS (China, Egypt, Ghana, India, Indonesia, Nigeria, Republic of Korea, Serbia, Singapore, South Africa, Thailand, United Republic of Tanzania, and Vietnam) (15), five in Africa were declared as having stable well-functioning integrated systems (maturity level 3 (ML3)), following formal benchmarking (BM) by a team of international experts of WHO. This remarkable achievement is the result of significant national, regional, and international investments and strong political commitment (16, 17).

Along with the African Medicines Regulatory Harmonization (AMRH) initiative in six regional economic communities (RECs) (18–24), WHO supports the harmonization of pharmaceutical regulatory policy for the benefit of NRAs (11) and their population (25), as well as the World Bank financially supported the initiative from US$12.5 million to approximately US$35.0 million over the last decade (26). Since 1975, the Economic Community of West African States (ECOWAS) has been established as a regional organization with 15 MS (Benin, Burkina Faso, Cape Verde, Cote d’Ivoire, Gambia, Ghana, Guinea, Guinea-Bissau, Liberia, Mali, Niger, Nigeria, Senegal, Sierra Leone, and Togo), with diverse political heritage which influence policies and business practices (27–29). In attaining the highest possible standard and protection of health in ECOWAS through the harmonization of health policies in MS, the West African Health Organization was created in 1987 as a specialized institution (30). With a population of approximately 365 million, a burden of heavy diseases (malaria, HIV/AIDS, tuberculosis, and neglected tropical diseases) and newly emerging diseases (31) occur in the region, confounding poverty and malnutrition and impacting the types of medicines needed. Differences in *lingua franca* (eight francophone countries, five Anglophone countries, and two lusophone countries) are reflected in the systems of regulation, further challenging medicine registration harmonization as a public health tool for improving access to quality medicines in the region (32, 33).

Since 1983, the Economic Community of Central African States (ECCAS) (34, 35) has been a regional organization with 11 MS (Angola, Burundi, Cameroon, Central African Republic, Chad, Congo, Democratic Republic of the Congo, Equatorial Guinea, Gabon, Rwanda, Sao Tome, and Principe) with different *lingua franca* (seven francophone countries, one anglo-francophone country, one hispanophone country, and two lusophone countries). These differences are also reflected in the system of medicine regulation. The harmonization of health policies is only active among six countries through the Organization for the Coordination of the Fight Against Endemic Diseases in Central Africa (OCEAC). Both ECCAS and ECOWAS are RECs recognized by the African Union (AU) (36).

Under WHO facilitation, the NRAs conduct self-benchmarking (SBM) to identify regulatory strengths and areas for improvement and elaborate institutional development plans (IDPs) to address gaps in a time-defined road map (11). Documented evidence of individual NRA performance identifying areas for improvement exists. However, cross-sectional analysis of SBM outcomes, highlighting common critical gaps to be addressed from regional perspectives, has not been systematically conducted and published. Limitations in comparing and replicating assessment results arise when an internationally harmonized tool, such as the GBT, is not utilized. Inconsistent and haphazard implementation plans hinder the timely and cost-effective achievement of stable well-functioning integrated regulatory systems (ML3), exacerbating inequities in access to quality-assured MPHT in LMICs (12). Moreover, the outcomes of the GBT at national, regional, and global levels have rarely been published in the literature.

The resulting complication is that to reach the desired ML, a rational, systematic, and reproducible method, taking into consideration common critical needs of the NRAs in a bottom-up top-down approach, has not been consistently applied. This results in missing opportunities to accelerate the targets of SDG 3.8 in a regionally coordinated and cost-effective manner (37). Thus, how can WHO GBT outcomes be used to identify the most common critical regulatory gaps and estimate the cost of addressing them so that the benefits of regional efforts towards achieving stable, well-functioning, and integrated systems (ML3) can be reaped within reasonable time?

WHO acts on evidence-informed approaches to achieve the triple billion targets for measurable impact on the health of people at country level, to better benefit from UHC, better protect from public health emergencies (PHE), and improve their health and well-being (38–40). The evidence on our cross-sectional study of WHO-facilitated SBM outcomes in 10 West and Central African countries indicates that concerted efforts on four critical pillars of regulation—preparedness for PHE (41), quality management system (QMS) (42, 43), control of substandard and falsified (SF) medical products (44, 45), and good regulatory practice (GRP)—could pave the way toward achieving ML3, thereby impacting access to quality-assured MPHT (46). Achieving ML3 by 2030 would improve the health and well-being of 203 million people in the 10 countries, and if replicated and expanded, 515 million people in the ECOWAS, 280 million people in the ECCAS, and 2 billion people in Africa (47).

The scientific methodology used in this study is reproducible by any REC, and the support by partners in international sustainable development would be catalytic. Regulatory system strengthening via regional coordination could also support the operationalization of a newly formed continental agency, the African Medicines Agency (AMA). The cost estimation power of the GBT can be instrumental for evidence-based managerial decision-making, fund raising, and advocacy by NRAs, RECs, and AMA. As studies using GBT outcomes continue to appear in peer-reviewed journals (41, 48–50), evidence supporting regional regulatory strengthening for increased access to assured-quality MPHT for all would accumulate.

We used the WHO-computerized GBT (cGBT) to collect and analyze data in a mixed method design. We performed a literature review to identify previous research related to the subject, ascertaining the gaps. The GBT outcomes of the 10 countries and the cost of interventions are presented by regulatory functions, ML, GBT sub-indicator categories, activity types, and RECs. The results are further dissected to pinpoint that most common gaps fall into four critical pillars of regulatory practice (PHE, QMS, GRP, and SF medical products) and are segregated by REC. At the end, we engage a conversation by recapping the gist and limitations of the study, pointing out contributions to regulatory science and proposing public health perspectives.

## 2 Materials and methods

### 2.1 Sample size

Through WHO-facilitated SBM, we collected data to evaluate the results of 10 countries, five ECCAS MS and five ECOWAS MS (11, 51, 52), one Spanish-speaking (Equatorial Guinea), and nine French-speaking (Cameroon, Chad, Gabon, Republic of Congo, Burkina Faso, Guinea, Ivory Coast, Niger, and Senegal). It is recognized that several *lingua franca* exist in the countries of the study; however, the language of the WHO African Region countries was used as the *lingua franca* for this study (52). The total population of these 10 countries in 2022 was estimated at 164 million (47). At the time of conducting the study, we chose these countries because their NRAs had completed their SBM, and full data were available. The WHO recommended that at least one NRA assessor per function performed the SBM. In keeping the confidentiality of countries, the results would be presented without linking the data to specific countries.

### 2.2 Data collection and strata

Through regional, on-line, and in-country WHO-facilitated SBM conducted between February 2020 and October 2021, we collected the data using the 196 WHO GBT published ML1 to ML3 sub-indicators (51) for 8 common regulatory functions for medicines comprising 9 GBT sub-indicator categories and 12 activity types (Table 1).

**Table 1:** Data collection through WHO-facilitated self-benchmarking events and strata by 8 common regulatory functions for medicines, 9 GBT sub-indicator categories, and 12 activity types for the 10 countries.

Benchmarking events	GBT common regulatory functions	GBT sub-indicator categories	Activity types
Face-to-face workshop, February 2020, Libreville, Gabon	National Regulatory System (RS)	Legal provisions, regulations, and guidelines	Equipment
In-country self-benchmarking, August to October 2021	Registration and Marketing Authorization (MA)	Organization and governance	Infrastructure
On-line self-benchmarking from April 2020 to September 2021	Vigilance (VL)	Policy and strategic planning	Workshop
	Market Surveillance and Control (MC)	Leadership and crisis management	Human resource (HR) recruitment
	Licensing Establishments (LI)	Quality and risk management system	Procedure elaboration
	Regulatory Inspections (RI)	Resources (human, financial, infrastructure, equipment)	Technical assistance
	Laboratory Testing (LT)	Regulatory process	Training
	Clinical Trial Oversight (CT)	Transparency, accountability and communication	Law formulation
		Monitoring progress and assessing outcomes, and impact	HR activities (performance evaluation, needs assessment, training planning and impact, job description elaboration)
			Information systems
			Communications
			Law enforcement

### 2.3 Institutional development plan elaboration and cost estimation

The 196 GBT sub-indicators were rated to establish their status of implementation and give a score for no implementation (0), partial implementation (0.25), on-going implementation (0.75), and complete implementation (1.0). National IDPs were elaborated by formulating recommendations to improve the not-fully implemented sub-indicators and/or maintain the fully implemented sub-indicators. The cost of implementing each recommendation was estimated using the approximate method, as shown in Table 2. We allowed for cost adjustment between countries, regions, and implementation partners. This costing approach has been used through hundreds of WHO BM and SBM exercises across the world. The IDP cost was estimated in US$ as per the time of the SBM between February 2020 and October 2021.

**Table 2:** Cost estimation (US$ between February 2020 and October 2021) of recommendations in institutional development plans.

Intervention	# Trainees / Item	Days	Estimated cost (US$)
In-country training	15	5	30,000
Technical support / consultancy	1	5	10,000
Overseas training	1	5	5,000
Job placement / secondment	1	5	5,000
Participation in regional workshop / meeting	1	5	3,000
Translation of essential documents – United Nations fees	1,000 words	–	240

### 2.4 Cutoff point for identification of common gaps

A total of 196 sub-indicators encompassing ML 1–3 in the eight common regulatory functions for medicines and nine GBT sub-indicator categories were used for analysis. As the target of SBM was medicines, the lot release function was excluded from the analysis. A cumulative score was estimated based on the grading of each of the 196 GBT sub-indicator ranging from 0 (no implementation), 0.25 (being implemented), 0.75 (partial implementation), to 1 (full implementation) (51).

**Figure 1:**
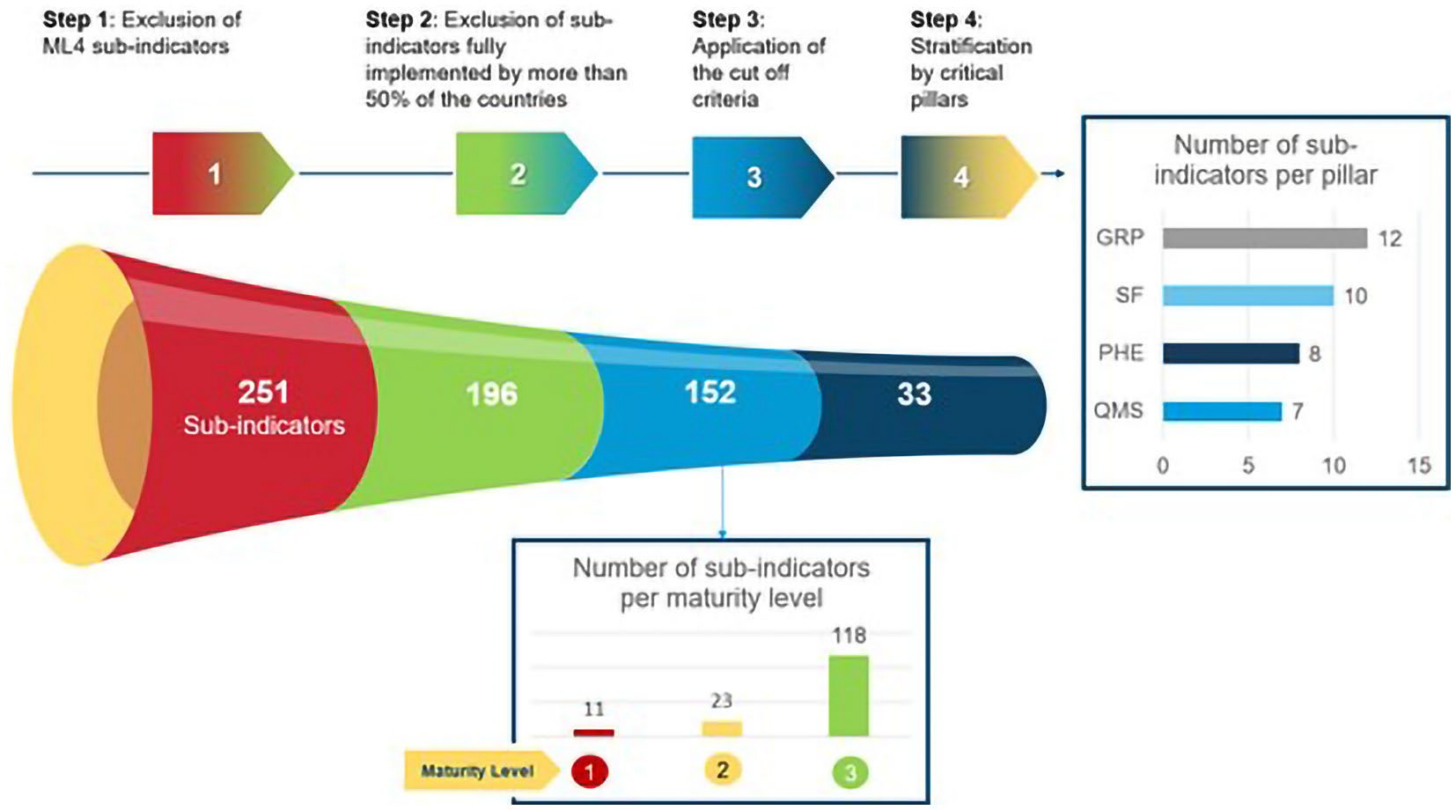
Methodology to establish the cutoff point to ascertain common regulatory gaps among GBT sub-indicators of eight regulatory functions for medicines up to maturity level 3 and four critical pillars of regulatory practice in the 10 countries.

Next, we identified the critical interventions that could fundamentally impact the road toward achieving ML3, in addition to estimating their cost. We established a cutoff point, considering only the 196 sub-indicators of the eight common regulatory functions for medicines up to ML3 and excluding the sub-indicators that were fully implemented by the majority (≥50%) of the countries. We further characterized the sub-indicators as “in need with high confidence” (sub-indicators not fully implemented in any of the countries or 0% implementation), ‘in need’ (sub-indicators not fully implemented in 50% or more of the countries or 10 to 50% implementation), and ‘not in need’ (sub-indicators implemented by more than 50% of the countries or > 50% implementation). The application of the cutoff criteria resulted in 152 sub-indicators (11 at ML1, 23 at ML2, and 118 at ML3) that were ‘in need with high confidence’ and ‘in need’ to be addressed (Figure 1). We considered these 152 sub-indicators as the ‘common gaps’ for further analysis.

### 2.5 Identification of common gaps in critical pillars of regulatory practice

We focused on identifying clusters among the common gaps that would fall into recognized critical areas of regulatory practice. We stratified the common gaps (152 sub-indicators) into two public health priorities: substandard and falsified (SF) medical products (53) and preparedness for PHE (41) and into two areas of NRA organizational efficiency: QMS (42, 43) and GRP (54). In this manner, 33 out of 152 GBT sub-indicators were identified as common gaps in the four pillars of regulatory practice and classified into ML 1 (red), 2 (yellow), and 3 (green) (Table 3).

**Table 3:** GBT sub-indicators (common gaps) relevant to quality management system (QMS), good regulatory practice (GRP), preparedness for public health emergency (PHE), and substandard falsified (SF) medical products across common regulatory functions and maturity levels (ML) 1 (red), 2 (yellow), and 3 (green) in the 10 countries.

Function	Sub-indicator	ML	QMS	GRP	PHE	SF
01—National Regulatory System	RS01.04: All regulatory entities (central and decentralized ones) follow non-contradictory regulations, standards, guidelines and procedures.	3		√		
	RS01.06: Legal provisions and regulations define requirements of transparency and dissemination of information to the public and relevant stakeholders.	2		√		
	RS01.09: A guideline on complaints and appeals against regulatory decisions is available to the public.	3		√		
	RS02.04: Independence of NRA from researchers, manufacturers, distributors and wholesalers, as well as from the procurement system.	2		√		
	RS03.04: Documented policies, procedures and mechanisms, including written criteria, are established for recognition and reliance on decisions of other NRAs (if applicable).	2			√	
	RS04.02: A rapid alert system for managing the threats by SF medical products and for recalling these products from the market.	2				√
	RS04.03: A rapid alert and recall system based on documented communication to the appropriate level of the distribution channel and with a feedback mechanism.	3				√
	RS04.04: Recall system based on documented confirmation that appropriate, batch-traceable action and/or destruction has been undertaken when necessary.	3				√
	RS04.05: Written criteria to cover circumstances in which the routine regulatory processes may not have to be followed in relation to crises and emergencies linked to a risk management plan.	3		√	√	
	RS05.01: Top management demonstrates commitment and leadership to develop and implement quality management system (QMS).	3	√			
	RS05.02: Quality policy, objectives, scope and action plans for establishment of the QMS are in place and communicated to all levels.	3	√			
	RS05.03: Organizational chart, with roles and responsibilities to establish the QMS are defined and in place.	3	√			
	RS05.04: Enough competent staff are assigned to develop, implement and maintain the QMS.	3	√			
	RS05.07: Requirements for documentation management as well as traceability of regulatory activities are established.	2	√			
	RS05.09: The externally provided products and services relevant to regulatory activities are controlled through established mechanisms.	3	√			
	RS05.11: Internal and external audits of the QMS are established and conducted at planned intervals.	3	√			
	RS09.04: Information on marketed medical products, authorized companies and licensed facilities is publicly available.	3		√		
	RS09.07: A code of conduct, which includes management of conflicts of interest, is published and enforced for internal and external staff, including members of the advisory committees.	3		√		
02—Registration and Marketing Authorization	MA01.06: There are legal provisions to cover circumstances under which the routine MA procedures may not be followed (e.g., for public-health interests)	1		√	√	
	MA01.12: There are established guidelines that cover circumstances under which the routine MA procedures may not be followed (e.g., for public-health interest)	3		√	√	
	MA04.06: Timelines for the assessment of the applications are defined and an internal tracking system has been established to monitor the targeted time frames	3		√		
	MA04.07: There are documented mechanisms to handle non routine registration and marketing authorization requirements in special situations (e.g., public-health interest)	3		√	√	
	MA05.02: Updated list of all medical products granted ma is regularly published and publicly available	3		√		
03—Vigilance	VL04.06: The NRA has access to expert committees for review of serious emergent safety concerns, when needed.	3			√	
04—Market Surveillance and Control	MC01.03: Legal provisions and/or regulations address the role of NRA in dealing with SF medical products.	1				√
	MC01.07: Guidelines exist on the recall, storage and disposal of SF medical products.	2				√
	MC04.04: Documented and implemented procedures exist for risk-based sampling of medical products from different points of the supply chain.	3				√
	MC04.05: Documented and implemented procedures exist to enable the public to report suspected SF medical products.	3				√
	MC04.07: Documented and implemented procedures and mechanisms exist to prevent, detect and respond to SF medical products.	3				√
	MC04.08: Documented and implemented procedures exist to ensure safe storage and disposal of detected SF medical products.	3				√
	MC06.03: Findings and regulatory decisions of market surveillance and control activities of common interest are appropriately communicated and shared with other countries and regional and international organizations.	3				√
06—Regulatory Inspections	RI01.05: Legal provisions and regulations allow the recognition of and/or reliance on foreign NRA inspections and enforcement actions based on well-defined criteria.	1			√	
08—Clinical Trial Oversight	CT01.11: Legal provisions and/or regulations allow the NRA to recognize and use relevant clinical trial decisions, reports or information from other NRAs, or from regional and international bodies.	1			√	

### 2.6 Cross-sectional analysis

The SBM results were cross-analyzed considering 196 GBT sub-indicators of eight common regulatory functions for medicines, maturity levels 1 to 3, 152 common gap sub-indicators, nine GBT sub-indicator categories, 12 activity types, 33 common gap sub-indicators in four pillars of regulatory practice, cumulative score of sub-indicator implementation, number of recommendations in the IDP, and estimated costs in US$ as per the time of the SBM between February 2020 and October 2021 per country and REC. As the analysis progressed, controls for bias and over representation in the data set were applied.

### 2.7 Software

The WHO-computerized GBT (cGBT, v12; v13) was used to assess the ML of regulatory systems for medicines. The Learning Management Software was used to produce the GBT training module. The NRA personnel were trained and certified on the use of the WHO cGBT. Microsoft Excel® was used for the analysis of SBM results and IDP data.

### 2.8 Literature review

A literature review query was conducted in PubMed using “strengthening AND regulatory AND system AND Africa AND medicines” as key words and Boolean operator. The search led to 73 publications which were segregated by mention and use of WHO GBT outcomes in national, regional, and global studies. Additional publications and WHO guidelines were added post co-author interviews. As no human subjects were used in this study, ethical considerations were not included.

## 3 Results

### 3.1 Maturity level and overall estimated cost of IDP recommendations

**Figure 2:**
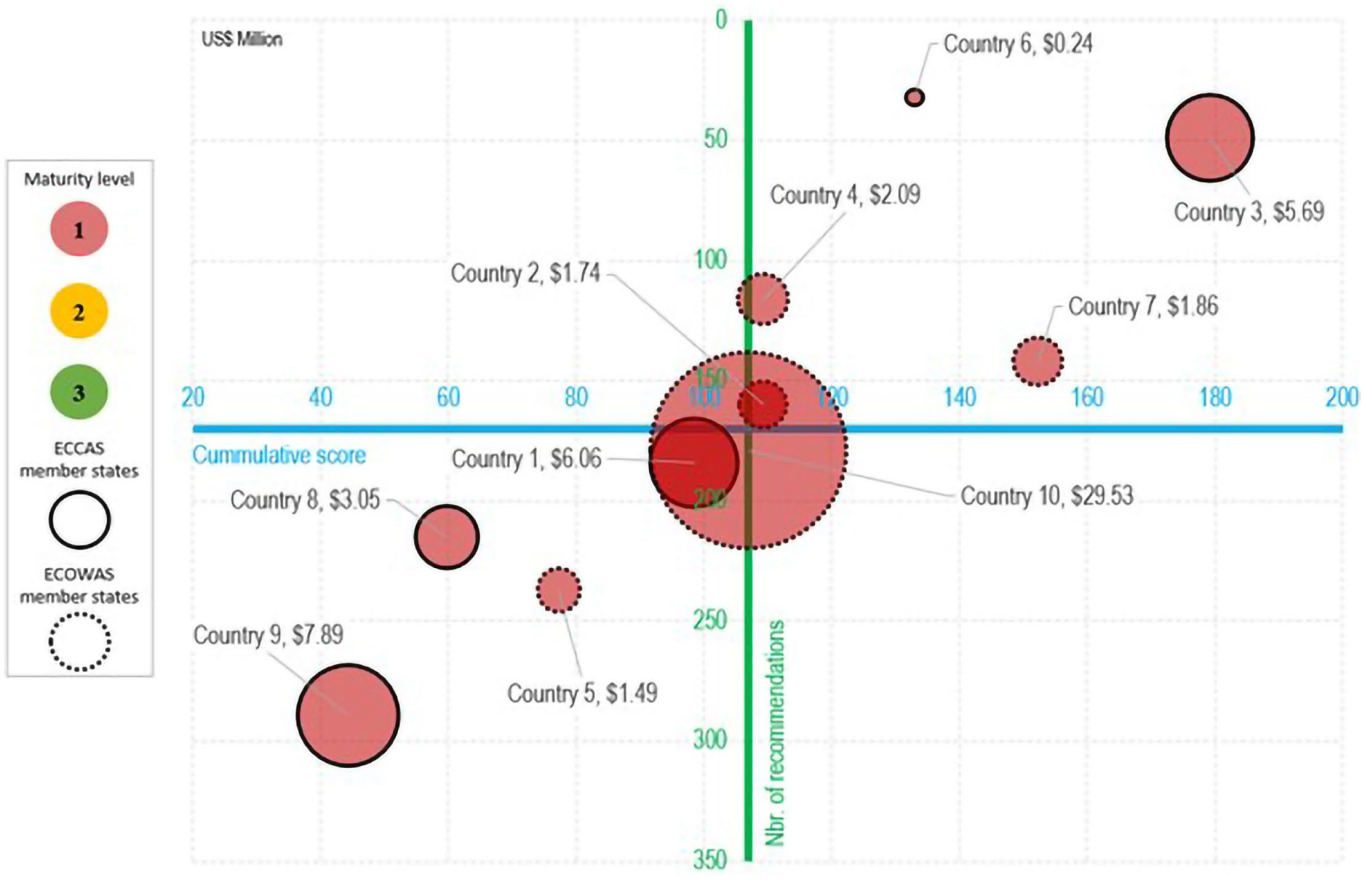
Estimated cost (US$ between February 2020 and October 2021) analysis of recommendations in the institutional development plans in eight common regulatory functions for medicines per maturity level, country, and regional economic community.

The cross-analysis of the SBM results revealed that the regulatory systems in the 10 countries were operating in a no-formal-to-reactive approach (ML1-ML2). The overall estimated cost of implementing 1,603 recommendations in the IDPs amounted to US$ 60 million, of which US$ 40 million (67%) were for equipment, HR recruitment, and infrastructure. The IDP cost of the five ECOWAS MS was estimated at US$ 36.7 million, while those of the five ECCAS MS was estimated at US$22.9 million (Figure 2).

**Figure 3:**
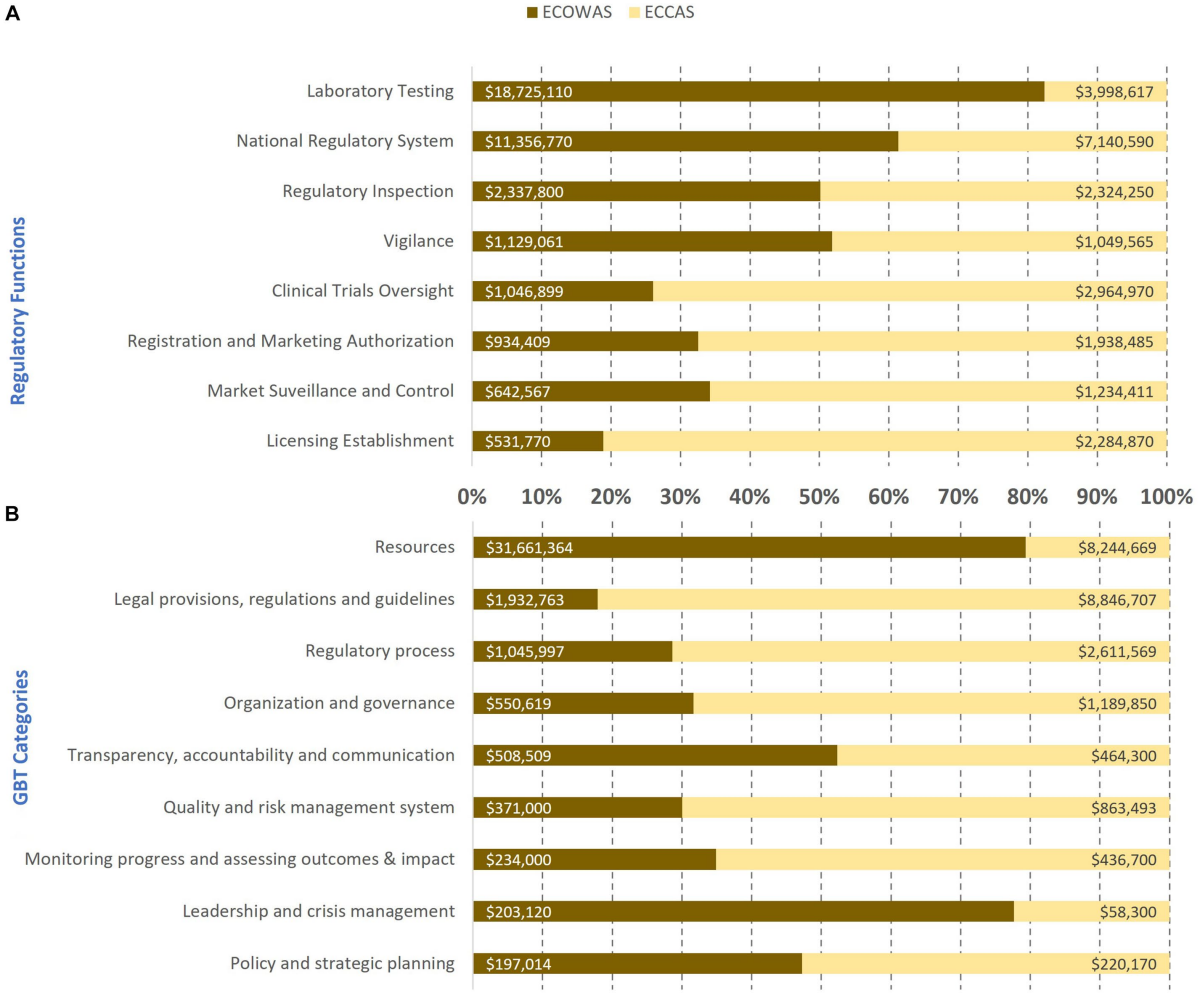
Estimated cost (US$ between February 2020 and October 2021) of the implementation of recommendations in institutional development plans per regulatory functions **(A)** and GBT sub-indicator categories **(B)** in 10 countries from two regional economic communities.

A high-level overview of the results is presented in a five-dimensional matrix that summarizes the cumulative score of sub-indicator implementation, number of recommendations in the IDP, and associated costs in US$ per country per REC. The average point for the cumulative score (107) and the number of recommendations (160) for the 10 countries are represented at the intersection of the two axes. The matrix displays four quadrants with countries distributed in three groups: countries 2, 3, 4, 6, and 7 (two ECCAS and three ECOWAS MS) in the upper right; countries 9, 5, 8, and 1 (three ECCAS and one ECOWAS MS) in the bottom left quadrant; and country 10 (ECOWAS MS) in the middle (Figure 3).

**Figure 4:**
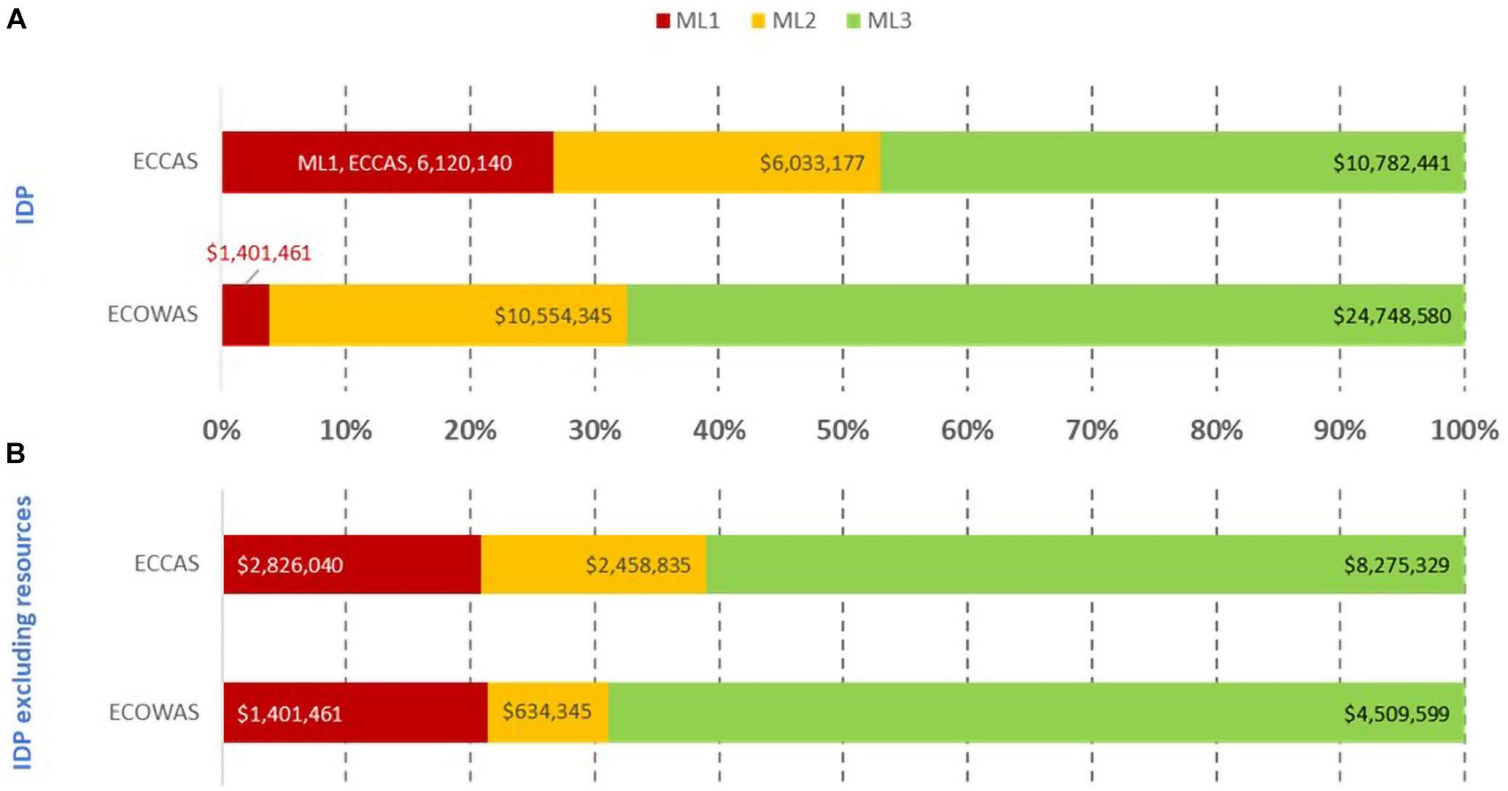
Estimated cost (US$ between February 2020 and October 2021) of recommendations in the institutional development plans of 10 countries including **(A)** and excluding resources [equipment, human resource recruitment, and infrastructure; **(B)**] per maturity level per regional economic community.

The estimated cost in US$ of 1,603 recommendations in the IDP was cross-analyzed and ranked per REC (Figures 3, 4). The estimated cost of recommendations in the IDPs of the 10 countries per regulatory function indicated that ECOWAS MS had the highest costing for laboratory testing followed by national regulatory system and vigilance, whereas in ECCAS MS, the highest costing fell on licensing establishments followed by clinical trials oversight, registration and marketing authorization, and market surveillance and control (Figure 3A). The horizontal bars represent the percentage of cost per regulatory function or GBT category per regional economic community with corresponding cost in US$ indicated inside each bar. The cross analysis of the estimated cost of IDP recommendations per GBT sub-indicator categories revealed that ECOWAS MS required higher investment in resources followed by leadership and crisis management, and transparency, accountability, and communication. In contrast, ECCAS MS bore higher costing for legal provisions, regulations, and guidelines followed by regulatory process, quality, and risk management system, organization and governance, monitoring progress and assessing outcomes and impact, and policy and strategic planning (Figure 3B).

Most of the IDP cost of addressing recommendations resided at ML3 in both RECs (Figure 4). The similar pattern was evident when including and excluding equipment, HR recruitment, and infrastructure in the analysis. When these resources were included, the IDP cost for ECOWAS was almost US$37 million, while for ECCAS, the IDP cost was US$23 million for US$60 million (Figure 4A). Once equipment, HR recruitment, and infrastructure were excluded, the IDP cost was US$ 6.5 million for ECOWAS and US$ 13.5 million for ECCAS for US$20 million (Figure 4B). The cumulative cost of addressing recommendations at ML1-ML2 in both RECs was US$24 million and US$7.3 million, including and excluding equipment, HR recruitment, and infrastructure, respectively. The cumulative cost of addressing recommendations at ML3 in both RECs was US$35.5 million and US$13 million, including and excluding these resources, respectively (Figure 4). The horizontal bars represent the percentage of cost per maturity level with corresponding cost in US$ indicated inside each bar.

### 3.2 Costing when excluding equipment, HR recruitment, and infrastructure

**Figure 5:**
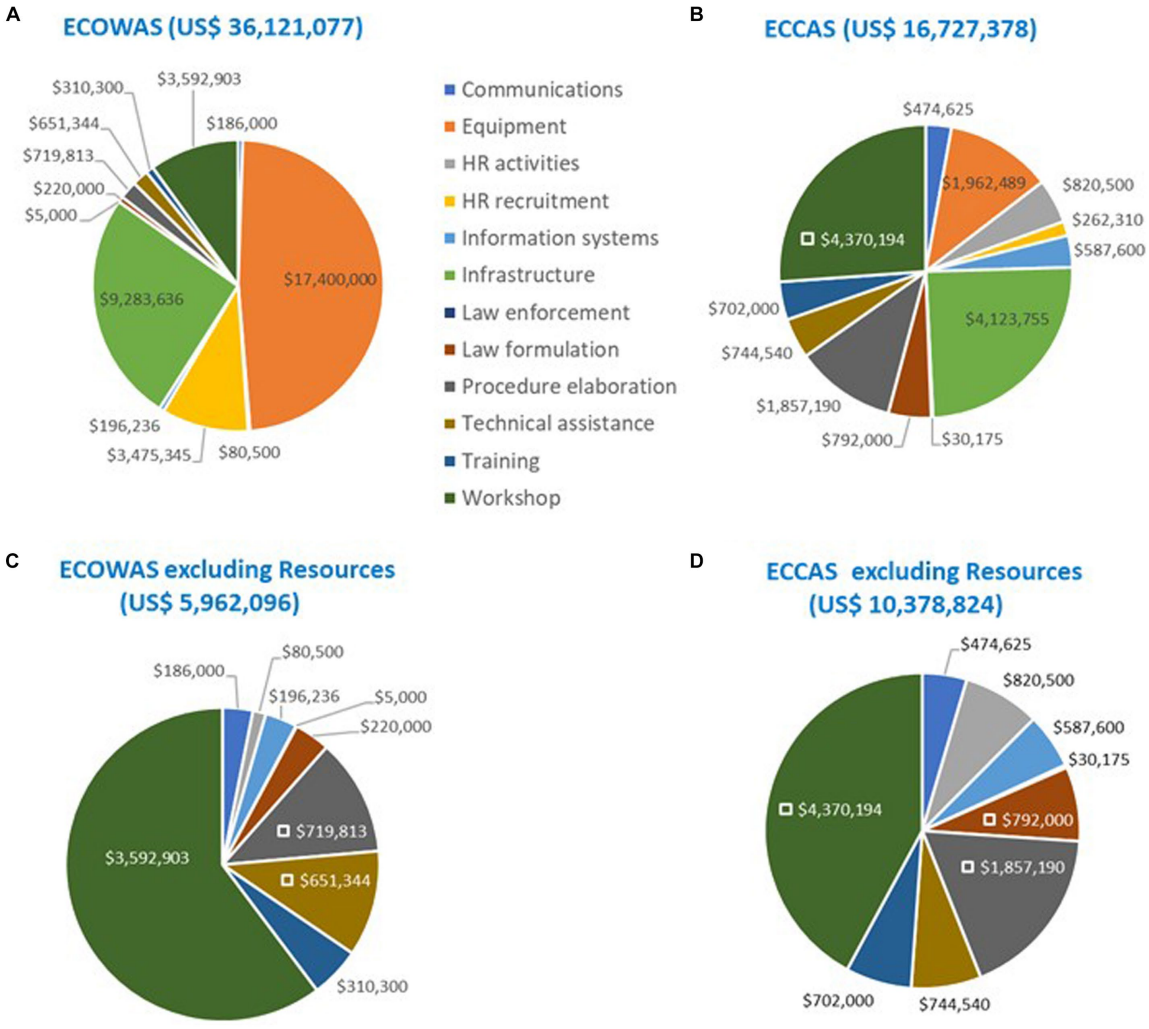
Estimated cost (US$ between February 2020 and October 2021) of recommendations in the institutional development plans of 10 countries to address common gaps by activity type and regional economic community including **(A,B)** and excluding “resources” [equipment, human resource recruitment, and infrastructure; **(C,D)**].

The analysis showed that addressing the common gaps (152 sub-indicators) in the IDPs of the 10 countries would cost an estimated US$53 million, including equipment, HR recruitment, and infrastructure, and US$16 million when excluding them. When examining the common gaps per 12 activity types per REC, the results confirm that equipment, HR recruitment, and infrastructure constituted the highest portion of the IDP cost for both ECOWAS and ECCAS MS. The overall cost of the IDP to address the common gaps, including these three resources, was US$36 million for ECOWAS and US$17 million for ECCAS (Figures 5A,B). Once the bias of the three resources was eliminated, the cost decreased to US$6 million for ECOWAS and US$10 million for ECCAS while revealing the most common activity types to address the gaps as workshops, followed by technical assistance, training, law formulation, law enforcement, procedure elaboration, information systems, HR activities, and communications (Figures 5C,D).

**Figure 6:**
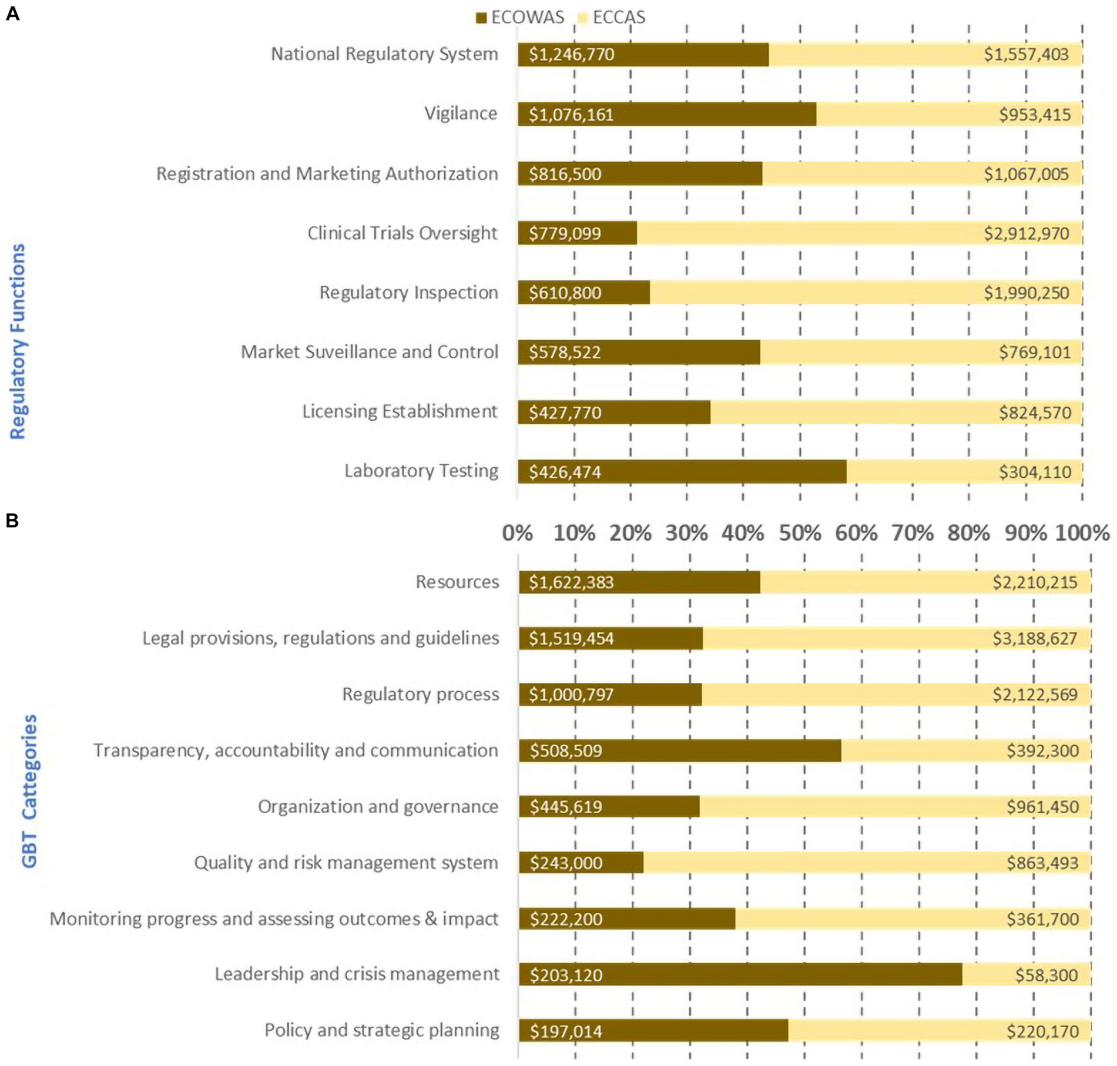
Estimated cost (US$ between February 2020 and October 2021) of recommendations in the institutional development plan to address common gaps per regulatory function **(A)** per GBT sub-indicator categories **(B)** per regional economic community excluding equipment, human resource recruitment, and infrastructure.

When analyzed per REC, the estimated cost of addressing common gaps for ECCAS MS was generally higher than the one for ECOWAS MS in six of eight common regulatory functions (Figure 6). ECOWAS MS had greater needs for laboratory testing and vigilance, whereas ECCAS MS had higher needs in clinical trial oversight, regulatory inspections, licensing establishments, market surveillance and control, and registration and marketing authorization (Figure 6A). The cross-analysis by GBT sub-indicator categories by REC indicated that, except for leadership and crisis management, transparency, accountability, and communications, ECCAS MS had greater needs in all other categories (Figure 6B). The horizontal bars represent the percentage of cost per regulatory function or GBT category per regional economic community with corresponding cost in US$ indicated inside each bar.

### 3.3 Cross-analysis of common gaps among the 10 countries

**Figure 7:**
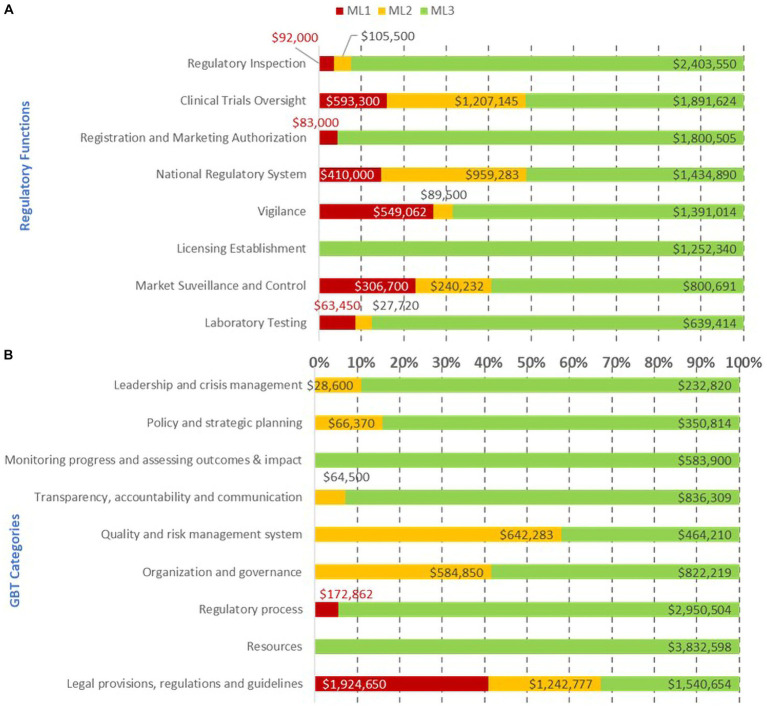
Estimated cost (US$ between February 2020 and October 2021) of recommendations in the institutional development plan to address common gaps by regulatory function **(A)**, GBT sub-indicator category **(B)**, and maturity level excluding resources (equipment, human resource recruitment, and infrastructure).

The cross-analysis of common gaps, excluding resources (equipment, HR recruitment, and infrastructure) between the 10 countries, led to an overall estimated cost of their IDPs per regulatory function and GBT sub-indicator categories per ML of US$16 million (Figure 7). The cumulative cost of addressing recommendations at a combination of ML1 and ML2 (US$4.2 million) was lower than in ML3 (US$12.7 million). The regulatory functions with the highest cost at ML1 and ML2 included, in decreasing order, clinical trial oversight, national regulatory system, vigilance, and market surveillance and control. The regulatory functions with the highest IDP cost, in decreasing order at ML3, included licensing establishments, registration and marketing authorization, regulatory inspections, laboratory testing, vigilance, national regulatory system, and clinical trials oversight. Clearly, most of the cost resided at ML3, which would be unattainable if the ML1-ML2 gaps were not addressed (Figure 7A). The horizontal bars represent the percentage of cost per maturity level with corresponding cost in US$ indicated inside each bar.

The cumulative cost of addressing gaps per GBT sub-indicator category at ML1-ML2 indicated that the legal provisions and regulations category demanded the highest investment followed by quality and risk management, organization and governance, policy and strategic planning, leadership and crisis management, transparency, accountability and communication, and regulatory process. The cost of addressing gaps at ML3 was greater than the combination of ML1-ML2 with investments needed in resources followed by monitoring progress and assessing outcomes and impact, regulatory process, transparency, accountability, and communication, leadership and crisis management, policy and strategic planning, and, finally, quality and risk management system (Figure 7B).

### 3.4 Common gaps found in four critical pillars of regulatory practice

Common gaps were revealed to fall into four critical pillars of well-functioning and integrated regulatory systems: QMS, GRP, PHE, and SF medical products. In total, 33 of 152 GBT sub-indicators encompassing ML 1–3 in six of eight common regulatory functions were identified as the common gaps in the four pillars of regulatory practice: QMS (7 sub-indicators), GRP (12 sub-indicators), PHE (8 sub-indicators), and SF medical products (10 sub-indicators), of which four sub-indicators (RS04.05, MA01.06, MA01.12, and MA04.07) overlapped the GRP and PHE pillars (Table 3).

**Figure 8:**
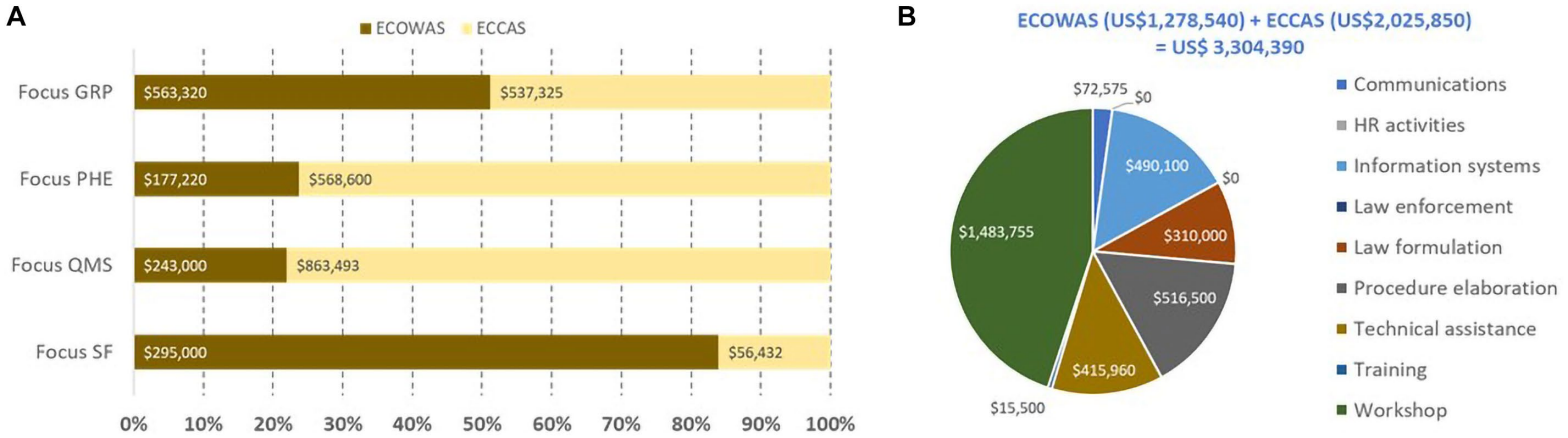
Estimated cost (US$ between February 2020 and October 2021) of recommendations in the institutional development plans of 10 countries to address common gaps in four pillars of regulation **(A)** per regional economic community per activity type [excluding equipment, human resource recruitment, and infrastructure; **(B)**].

The overall estimated cost of addressing common gaps in four critical pillars of well-functioning and integrated regulatory systems was US$3.3 million, of which US$2 million and US$1.3 million were for ECCAS MS and ECOWAS MS, respectively (Figure 8). The estimated cost of addressing the common gaps was US$1.1 million, US$0.74 million, US$1.1 million, and US$0.35 million for GRP, PHE, QMS, and SF medical product pillars, respectively. The highest cost of addressing common gaps in GRP and SF medical products was observed in ECOWAS MS, while for PHE and QMS, the highest cost of addressing common gaps was observed in ECCAS MS (Figure 8A). The cost distribution per pillar of regulation per activity type indicated that the most common activity chosen to address gaps was workshops, followed by technical assistance, procedure elaboration, law formulation, information systems, communications, and training (Figure 8B). The horizontal bars (A) represent the percentage of cost per pillar per regional economic community with corresponding cost in US$ indicated inside each bar (B). The pie graph represents the activity types to address common gaps in the four pillars with corresponding cost in US$ indicated inside each bar (B).

**Figure 9:**
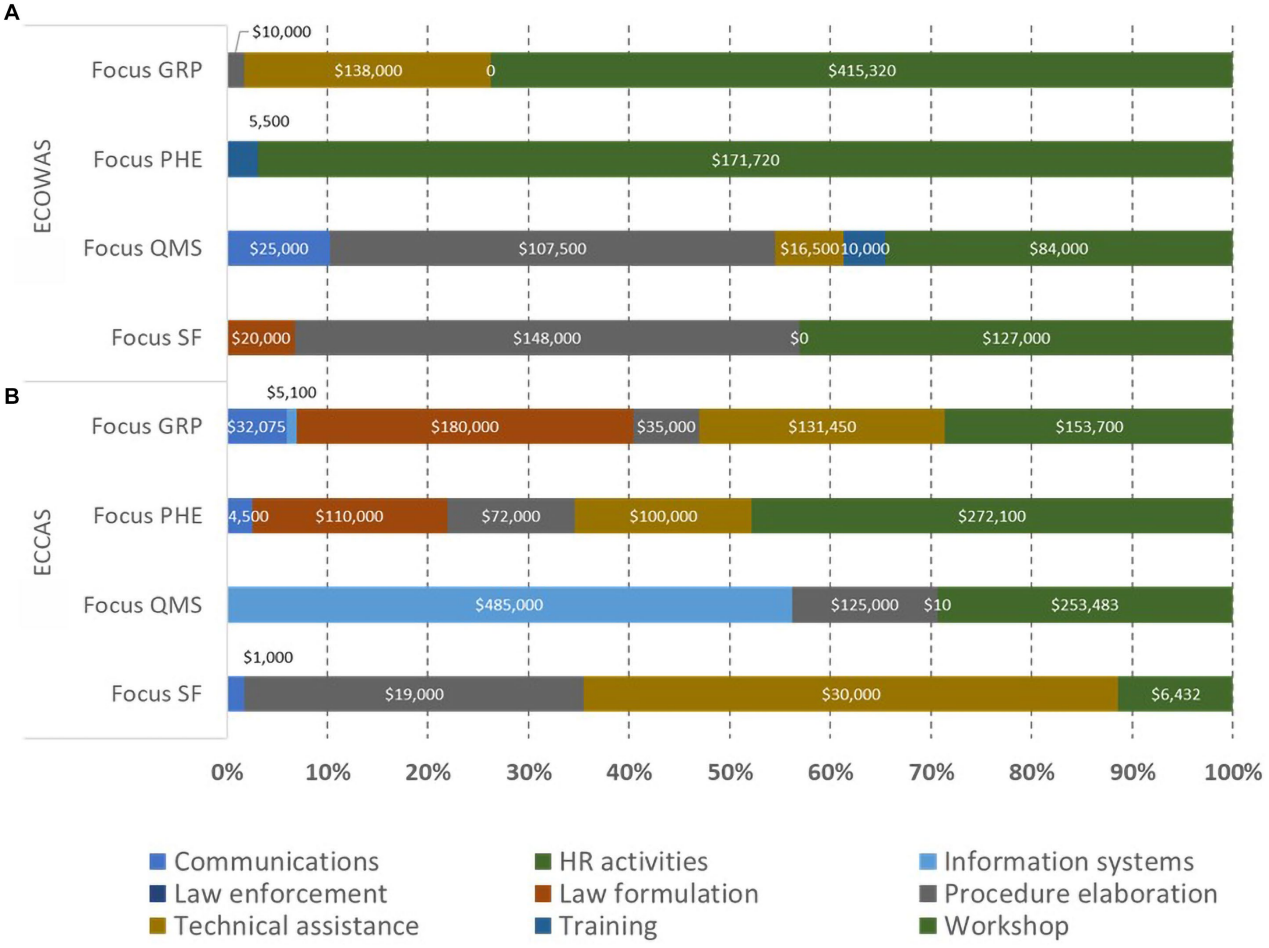
Estimated cost (US$ between February 2020 and October 2021) of recommendations in the institutional development plans of 10 countries to address common gaps in four pillars of regulation by activity types per regional economic community [**(A)**—ECOWAS, **(B)**—ECCAS].

The distribution of activity types per pillar of regulation per REC indicated that ECOWAS MS would address the common gaps mostly via workshops and technical assistance for the GRP and PHE pillars; workshops, procedure elaboration, technical assistance, communication, and training for the QMS pillar; and workshops, procedure elaboration, and law formulation for the SF medical products pillar (Figure 9A). ECCAS MS would address the common gaps via workshops, technical assistance, procedure elaboration, law formulation, information systems, and communications for the GRP pillar; workshops technical assistance, procedure elaboration, law formulation, and communications for the PHE pillar; information systems, workshops, procedure elaboration for the QMS pillar; and, finally, technical assistance, procedure elaboration, workshops, and communications for the SF medical products pillar (Figure 9B). The horizontal bars represent the percentage of cost per pillar with corresponding cost in US$ indicated inside each bar.

**Figure 10:**
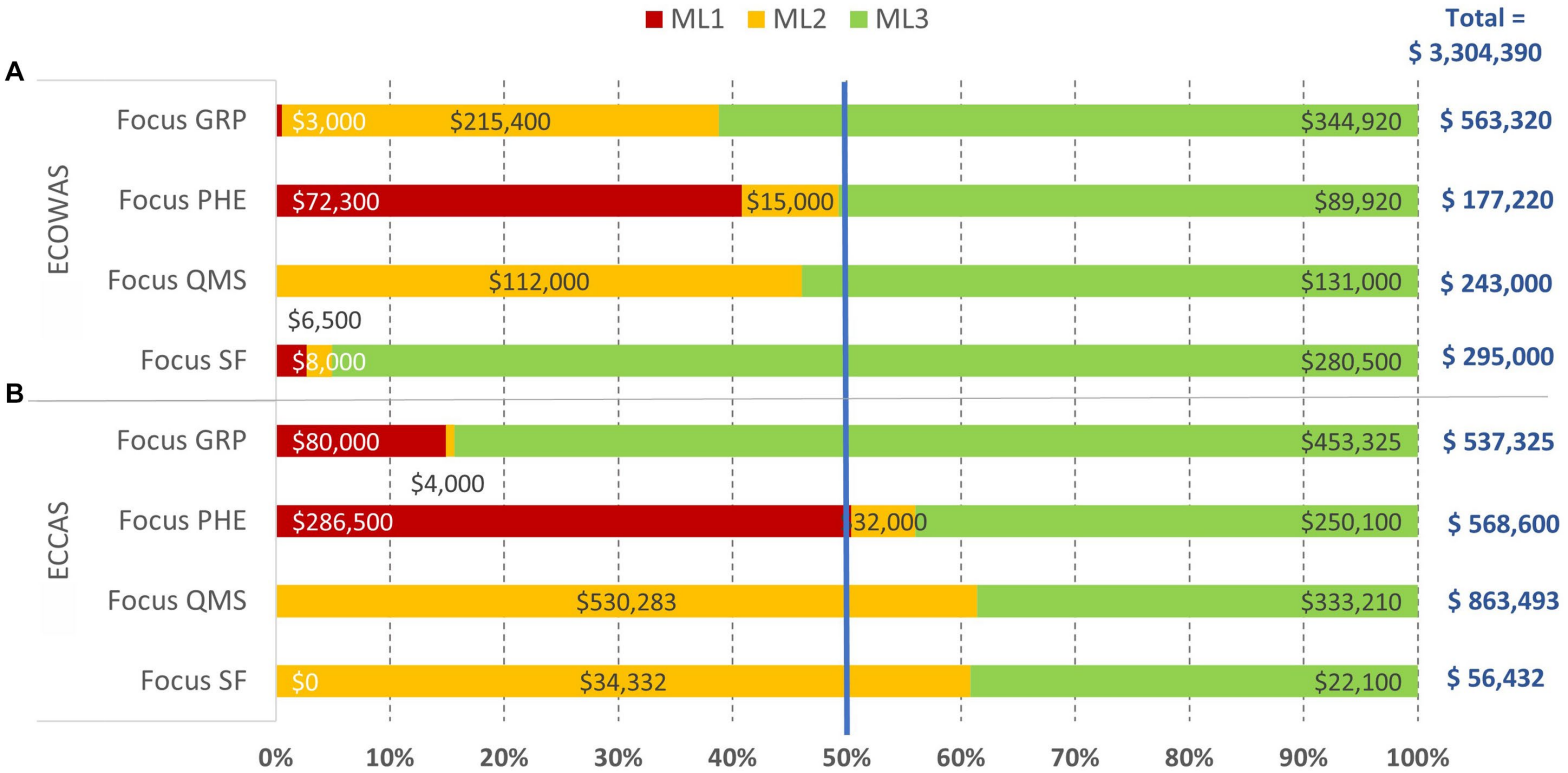
Overall estimated cost (US$ between February 2020 and October 2021) of recommendations in the institutional development plans of 10 countries to address common gaps in the four pillars of regulation, excluding equipment, human resource recruitment, and infrastructure, per maturity level per regional economic community [**(A)**—ECOWAS, **(B)**—ECCAS].

The estimated cost of addressing common gaps in each of the four pillars per ML per REC is shown in Figure 10. Most of the cost of addressing common gaps were at ML3 for both ECOWAS MS and ECCAS MS; however, the foundational gaps resided at ML1-ML2. For ECCAS, ML1 and ML2 combined investments were needed to address common gaps in PHE (US$318,500; 56%) and GRP (US$84,000; 16%). In contrast, only recommendations at ML2 needed to be addressed for QMS (US$530,283; 61%) and SF medical products (US$34,332; 61%). For ECOWAS, ML1 and ML2 combined investments were needed to address gaps in GRP (US$218,400; 39%) and PHE (US$87,300; 49%). Only recommendations at ML2 needed to be addressed for QMS (US$112,000; 46%) and SF medical products (US$14,500; 5%). The overall cost of addressing common gaps in the four pillars was US$1.3 million for a combination of ML1 and ML2 and US$2 million for ML3 in both ECOWAS MS and ECCAS MS (Figure 10). The horizontal bars represent the percentage of cost per maturity level with corresponding cost in US$ indicated inside each bar.

## 4 Discussion

### 4.1 Key findings

In this study, we used the peer-developed internationally standardized data gathering tool, the WHO cGBT, to identify common gaps in national regulatory systems for medical products, estimate the cost of addressing gaps, and develop evidence-based solutions that can be coordinated via REC approaches. Cost estimation of recommendations in the IDPs was done in US$ as of February 2020 to October 2021. Our analysis of overall IDP cost points to specific countries as “low hanging fruits” for immediate investment, while others would require significantly higher investment to reach the same level.

We highlighted evidence-based improvements in four critical pillars of regulation (GRP, PHE, QMS, and SF medical products), to inform managerial decision-making and catalyze the transition of regulatory systems from ML1-ML3 in eight common regulatory functions for medicines across five ECCAS and five ECOWAS MS. Furthermore, we propose solutions on where and how to invest and what regional approaches to take to address common gaps.

We estimated that US$20 million investment would help push the 10 countries into ML3 in all regulatory functions within reasonable time. The cost of addressing the most common gaps was estimated at US$16 million, which can be further reduced to US$3.3 million by focusing on the four critical pillars. The estimated cost of IDP to address common gaps in the four pillars for ECCAS and ECOWAS MS was US$2 and US$1.3 million, respectively.

This represents an evidence-based managerial decision-making point. WHO recommends addressing gaps at ML1 and then ML2 and ML3 (11); thus, managers could opt to implement recommendations to address common gaps at ML1 in a ´low-hanging fruit’ concept. In this context, ECOWAS would address gaps in PHE, SF, GRP, and QMS, sequentially, while ECCAS would address gaps in PHE, GRP, QMS, and SF, sequentially (Figure 10). If availability of funds was the limiting factor, the managerial path would be to address recommendations at the lowest cost. In this manner, ECOWAS would address PHE, QMS, SF, and GRP, whereas ECCAS would address SF, GRP, PHE, and QMS, sequentially. Whichever the context might be, it is evident that in three of the four contexts above, PHE comes as the main priority to be addressed (Figure 10).

Our study offers a concrete foundational plan for improving regulatory systems in the 10 countries with potential for replication in other RECs. By investing in the four critical pillars, these countries can strengthen their regulatory systems at a foundational level and improve access to quality, safe, and effective medical products for their populations within reasonable time.

### 4.2 Prior research related to the subject

Information on GBT assessment outcomes available in the public domain is limited. WHO GBT assessment outcomes have directly been used in merely two articles at regional level (48, 49), one at global level (41) and another one at national level (50).

In 2012, the WHO Data Collection Tool for the Review of Drug Regulatory Systems, a predecessor of the GBT, was used jointly with a practical guidance in the ECCAS and OCEAC regions (48), to document gaps in the regulatory systems of nine West and Central African countries. Although concrete ways to address the gaps were presented, the cost of implementation was not estimated. In our 2020–2021 study, the NRAs in 10 countries, also in Central and West Africa, were assessed using the WHO cGBT, and the cost of implementing IDP recommendations was estimated. Nonetheless, the 2012 study and our study agree in the common gaps and the need to address them in collaboration with the RECs.

At a global level, Broojerdi et al. (41) documented the gaps and challenges on regulatory preparedness for PHE using WHO GBT outcomes from 84 MS and proposed strategic, harmonized, and sustainable regulatory solutions to prepare for PHE. At national level, Shabani et al. (50) published the GBT outcomes of the Rwanda Food and Drug Authority with the identification of gaps and existing opportunities to improve regulatory capacity that ensure the quality of medicines (39).

In a cross-sectional study carried out by state and non-state actors, Samukange et al. (49) introduced the concepts of “cluster,” the “least implemented sub-indicators,” and the “medium score” by a 13-step method using the WHO GBT plus Blood when assessing the hemovigilance function of NRAs in 10 EAC, ECOWAS, and SADC MS (38). Similarly, we introduce the concept of ´common gaps´, and, like the methodology by Samukange et al., our concept is also reproducible.

The RECs in Africa are increasingly publishing their work with MS and common interest, often with different approaches. In a 2020 study of drug safety and surveillance (55) in the EAC, internally harmonized pharmacovigilance and GBT sub-indicators were used to assess strengths and limitations of the national pharmacovigilance systems of four countries, one of which had already reached ML3 for medicines. Identifying common gaps in a REC at different MLs while using complementary tools to the GBT aided in assessing potential skewedness in pharmacovigilance outcomes.

The Caribbean Community and Common Market illustrates a regional approach to empower small nations to group up with others with common *lingua franca*, culture, history, and economic conditions to work together to make regulatory systems more efficient (46). This case study supports the notion of building and establishing basic regulatory capacities that meet public health needs, simultaneously ensuring regional sustainability. In 2019, the WHO regional office for South-East Asia reported on the regulatory status, highlighting the suitability of GBT outcomes for the identification of regional common gaps and collaboration in addressing them (3).

The main WHO BM products are a country-specific IDP and a time-bound road map toward achieving ML3 with limited consideration for regional coordination and regulatory harmonization included in the plan (2). WHO GBT outcomes have been used partially in several studies at regional level often explaining the tool, yet not exploring further its added value at regional level. Moreover, how are studies to follow a common and easily reproducible method to collaborate and coordinate regulatory systems strengthening at regional level?

Our scientific results support the notion that well-documented BM outcomes would serve to strengthen national and regional institutions and operations, to become official agencies that have already planned to set their legal mandate (17, 44), thereby catalyzing equitable access to quality-assured medical products. In this regard, the WHO GBT platform could be used to systematically define common needs in any given REC to develop and implement common solutions (56).

### 4.3 Limitations of the study

The WHO GBT was used as a financial tool rather than a tool to conduct value for money assessment; however, according to Guzman et al. (52), it could be strengthened to provide a more rigorous methodology for estimating costs of IDP implementation. Nonetheless, we maximized the use of the GBT to estimate an approximate cost of IDP that can support managerial decision-making, advocacy, and fund raising at national, regional, and global levels. The WHO GBT was not designed as a financial tool; however, according to Guzman et al. (52), it could be strengthened to provide a more rigorous methodology for estimating costs of IDP implementation. Despite its limitations, we used the GBT to estimate an approximate cost of IDP that can support managerial decision-making, advocacy, and fund raising at national, regional, and global levels.

Our study covers only regulation of medicines and not medical devices, vaccines, blood, and blood products in the 10 countries, mostly francophone, in sub-Saharan Africa. Furthermore, the data were generated through WHO-assisted SBM, which constitutes one of several steps toward the formal BM by a team of international experts (11). In addition, this study was conducted during the COVID1-9 pandemic, which posed significant constraints for SBM activities and data acquisition. Internet connections were weak making on-line SBM painfully time- and effort-consuming (Table 1).

Equipment, HR recruitment, and infrastructure must undoubtedly be adequate for the implementation of IDP recommendations to address gaps. Unfortunately, the estimated cost of these activities is missing in 8 out of the 10 countries in the data set. The operational cost of IDP implementation by the NRA and the financial and technical partners is not considered in the estimates. These operational costs may vary from the IDP cost estimation methodology (Table 2) by institution, implementing partner, country, region, and other factors.

As the study was conducted in 2020–2021 and countries have already started implementing recommendations in their IDPs, sub-indicator scores and cost of addressing gaps might have changed. Countries are also recovering from the hardships of the COVID-19 pandemic. It could also be expected that as the NRAs become more mature and more independent from the MoH, their budgets would become more granular, particularly on market surveillance and control budgets which would become more granular. Thus, our findings constitute a baseline for NRAs and RECs, moving forward using the four critical pillars of regulation and the concept of common gaps at regional level. As this study includes five MS of each ECCAS and ECOWAS, it represents only a partial appreciation of actual regulatory gaps in these RECs. Thus, regulatory systems strengthening of medical products could be supported via regional approaches by benchmarking NRAs using the WHO GBT and addressing common gaps in the four critical pillars in all MS in the RECs.

### 4.4 Integration of key findings into current regulatory thinking

The bottom-up evidence-based recommendation is that to maximize impact on in-country access to quality, safe, effective, and affordable medical products, an approach focusing on well-functioning and integrated regulatory systems should include, at minimum, preparedness for PHE, implementation of QMS, control of SF medical products, and implementation of GRP. The current understanding is that, with strong leadership commitment, IDP recommendations at ML1-2 should be addressed for solid arrival in ML3 within reasonable time. Our view is that addressing common gaps in the four critical pillars at ML1-2 (10 GBT sub-indicators) and then ML3 (23 GBT sub-indicators) (Table 3) would strengthen, at a foundational plane, the ability of a country to ensure the quality of medical products and response to public health emergencies, e.g., pandemics, in a timely manner.

Our proposed solution for a regulatory system, where these common gaps are addressed in a systematic and pragmatic (ML1 to ML2 and ML3) manner for each of the pillars, is presented in Table 3.

For the GRP and PHE pillars at ML1, legal provisions covering circumstances under which routine marketing authorization procedures may not be followed to address public health emergencies should be formulated (MA01.06). For the GRP pillar at ML2, legal provisions and regulations that define requirements of transparency and dissemination of information to the public and relevant stakeholders should be formulated (RS01.06), while independence of the NRA from researchers, manufacturers, distributors, wholesalers, and the procurement system should be ascertained (RS02.04).

In preparation for PHE (41) (9 sub-indicators) at ML1, based on well-defined criteria, legal provisions and regulations that allow the recognition of and/or reliance on foreign NRA inspections and enforcement actions should be established (RI01.05). Similarly, legal provisions and/or regulations that allow the NRA to recognize and use relevant clinical trial decisions, reports, or information from other NRAs or from regional and international bodies should be in place (CT01.11). For preparedness for the PHE pillar at ML2, policies, procedures, and mechanisms, including written criteria, should be documented to recognize and rely on the decisions of other NRAs (RS03.04). The GBT does not include sub-indicators at ML1 for the QMS pillar; however, requirements for documentation management and traceability of regulatory activities should be established for ML2 (RS05.07).

In the realm of SF medical products pillar at ML1, legal provisions and/or regulations addressing the role of the NRA in dealing with SF medical products (MC01.03) should be formulated. The existence of a rapid alert system for managing the threats by SF medical products and recalling these products from the market (RS04.02), as well as guidelines on the recall, storage, and disposal of SF medical products (MC01.07), would address common gaps at ML2.

Once common gaps at ML1 and ML2 have been addressed for each of the pillars, our proposed solution for those at ML3 is presented in Table 3.

For the GRP and PHE pillars overlap at ML3, written criteria to cover circumstances, in which the routine regulatory processes may not be followed due to crises and emergencies, should be established and linked to a risk management plan (RS04.05). Guidelines covering circumstances under which the routine MA procedures may not be followed (e.g., for public-health interest) (MA01.12) should be implemented, while mechanisms to handle non-routine registration and MA requirements in special situations (i.e., public-health interest) (MA04.07) should be thoroughly documented. In addition, for the PHE pillar at ML3, having access to expert committee for review of serious emergency safety concerns, when needed (VL04.06), should be ensured.

For the QMS pillar at ML3, the top management should have demonstrated commitment and leadership to develop and implement QMS (RS05.01). A quality policy, objective, scope, and action plans for establishment of the QMS should be in place and communicated to all levels of the organization (RS05.02). An organizational chart, with roles and responsibilities to establish the QMS, should be defined and in place (RS05.03) with enough competent staff assigned to develop, implement, and maintain the QMS (RS05.04). Finally, mechanisms to control externally provided products and services that are relevant to regulatory activities should be in place (RS05.09), while internal and external audits of the QMS should be established and conducted at regularly planned intervals (RS05.11).

For the SF medical products pillar at ML3, based on documented communication to the appropriate level of the distribution channel and with a feedback mechanism, a rapid alert and recall system should exist (RS04.03). The recall system would be based on documented confirmation that appropriate, batch-traceable action and/or destruction was undertaken, when necessary (RS04.04). Documented and implemented procedures for risk-based sampling of medical products from different points of the supply chain (MC04.04) would exist. Furthermore, documented and implemented procedures should also exist to enable the public to report suspected SF medical products (MC04.05) and prevent, detect, and respond to SF medical products (MC04.07). Finally, documented and implemented procedures would be established to ensure safe storage and disposal of detected SF medical products and control activities of common interest appropriately communicated and shared with other countries and regional and international organizations (MC04.08).

As per the implementation of GRP pillar at ML3, all regulatory entities (central and decentralized ones) would follow non-contradictory regulations, standards, guidelines, and procedures (RS01.04). In addition, a guideline on complaints and appeals against regulatory decisions would be available to the public (RS01.09), while information on marketed medical products, authorized companies, and licensed facilities should also be publicly available (RS09.04). Furthermore, a code of conduct, which includes management of conflicts of interest, would be published and enforced for internal and external parties, including members of advisory committees (RS09.07). Timelines for the assessment of the applications would be defined, and an internal tracking system would be established to monitor targeted time frames (MA04.06). Finally, an updated list of all medical products for which MA was granted would regularly be published and publicly available (MA05.02).

In summary, our proposed solution for a system to address common gaps in each of the four critical pillars of regulation implies resilient legal provisions and regulations, informed reliance on decisions of other regulatory bodies (46, 57), and collaboration among national, regional, and global partners with transparency, accountability, and communication. We also proposed that a concerted investment of US$ 3.3 million (Figure 10) for activities to address common gaps in the four critical pillars of regulatory practice, ideally driven by collaborating regional interventions, would maximize the impact on the 10 countries at a foundational level.

### 4.5 Regulatory science perspectives

This study draws attention to the need for rational, systematic, and fundamental regulatory capacity building, not only in English-speaking African countries but also in countries with French, Spanish, and Portuguese as *lingua franca*. The study lays out a comprehensible methodology to identify common gaps and estimate the cost of addressing them regionally, which can be replicated by others, thereby expanding the benefits to other RECs.

In the spirit of leaving no one behind, the use of the GBT should expand to languages that the users know best. As an educated international RSS community with proficient GBT users continues to grow, so would the number of assessors in languages other than English. An increased number of proficient assessors in French, Spanish, and Portuguese languages would result in improved scientific documentation of regulatory functioning, BM knowledge acquisition, evidence gathering, capacity building, and decision-making in LMIC.

We envision the cross analysis of all BM results from African NRAs conducing to elaborating IDP implementation strategies for collaboration between the AMRH, RECs, AMA, and others. This methodology can be integrated into their roadmaps and be published as follow-up studies. Our vision involves elaborating and publishing a 5–7-year implementation plan focusing on the four critical pillars of well-functioning and integrated regulatory systems to document and monitor improvements in the 10 countries. This process would necessarily imply the involvement of the ECCAS and ECOWAS harmonization programs, international technical and financial partners, and the countries’ willingness to share BM findings below ML3. This call for openness should be backed by adequate political, leadership, and funding commitment, focusing on LMIC.

Unless the NRA achieves ML3, BM outcomes are rarely shared, thus evidence-based and experience-based information goes unpublished (10). Exceptionally, the Rwanda Food and Drug Authority (FDA) in collaboration with the University of Rwanda published assessment results at ML2 and shared the challenges, hindering the implementation of key regulatory functions in their journey toward ML3 (50). In contrast, Ghana (58) and Tanzania (59) shared their knowledge and experience after achieving ML3. In emulating Rwanda FDA and the spirit of contributing to regulatory science, opening access to the GBT for academic and public health researchers would improve knowledge and experience sharing, regardless of the ML (10). Opening access to the GBT outcomes through the CIP membership would offer scholars and other non-state actors in Africa with opportunities to collaborate, research, and publish findings, irrespective of the ML. Currently, there are no African universities as members of the CIP (60).

The extended use of the GBT has generated a regulatory momentum in Africa with several NRAs, achieving ML3 status in the last quinquennium (Egypt, Ghana, Nigeria, South Africa, and United Republic of Tanzania) (15). In a kind of ‘early warning system’, monitoring implementation of the common-gap sub-indicators could signal the minimum requirements to protect the population from harm by unsafe MPHT. The capacity built in these countries, mostly anglophone countries in Africa, has impacted national and regional harmonization. Even though BM activity in countries with *lingua franca* French, Spanish, and Portuguese has increased, much remains to be done to expand the benefits of regulatory systems strengthening to Central and West Africa, including all the 11 ECAAS and the 15 ECOWAS MS.

Funding aspects of regulatory systems strengthening are highlighted in this study with the perspective that the cost of targeted interventions to address common gaps can be estimated using the GBT, to attract financial and technical partners using existing REC harmonization initiatives, thereby maximizing the impact. It is expected that addressing gaps in the four critical pillars will strengthen regulatory reliance for all medical products, which could also be REC-based, including all countries in Central and West Africa.

## 5 Conclusion

We identified common gaps in four critical regulatory pillars in 10 West and Central African countries to strengthen national regulatory systems and promote equitable access to medical products. By addressing these gaps and leveraging REC harmonization initiatives, regulatory reliance can be improved for all medical products in LMICs. Follow-up studies are needed to expand the impact to other countries in the region. The AMA offers momentum to leave no one behind and address historical inequities in pharmaceutical regulation. Well-funded medicine regulatory harmonization agencies within the RECs would support the operationalization and sustainability of AMA.

## Data availability statement

The raw data supporting the conclusions of this article will be made available by the authors, without undue reservation.

## Author contributions

CA: Conceptualization, Formal analysis, Investigation, Methodology, Supervision, Validation, Visualization, Writing – original draft, Writing – review & editing. GN’J: Conceptualization, Data curation, Formal analysis, Investigation, Methodology, Project administration, Software, Visualization, Writing – original draft, Writing – review & editing. AM: Methodology, Software, Writing – review & editing¸ Formal analysis. LT: Writing – review & editing, Investigation. RK: Writing – review & editing. AK: Writing – review & editing. DM: Writing – review & editing. SO-A-Y: Writing – review & editing. AD: Writing – review & editing. MN-S: Writing – review & editing.
